# Identification of long noncoding RNAs in injury-resilient and injury-susceptible mouse retinal ganglion cells

**DOI:** 10.1186/s12864-021-08050-x

**Published:** 2021-10-14

**Authors:** Ana C. Ayupe, Felipe Beckedorff, Konstantin Levay, Benito Yon, Yadira Salgueiro, Ramin Shiekhattar, Kevin K. Park

**Affiliations:** 1 Department of Neurological Surgery, Miami Project to Cure Paralysis, University of Miami Miller School of Medicine, 1095 NW 14th Ter, FL 33136 Miami, USA; 2 Sylvester Comprehensive Cancer Center, Department of Human Genetics, Biomedical Research Building, University of Miami Miller School of Medicine, Room 719, 1501 NW 10th Avenue, Miami, FL 33136 USA

**Keywords:** Axon injury, Retinal ganglion cell, Axon regeneration, lncRNAs, Retina, Intrinsically photosensitive RGCs, Direction selective RGCs, Neuronal type specification, Neuronal survival, Neuronal apoptosis

## Abstract

**Background:**

Emerging evidence indicates that long noncoding RNAs (lncRNAs) are important regulators of various biological processes, and their expression can be altered following certain pathological conditions, including central nervous system injury. Retinal ganglion cells (RGCs), whose axons form the optic nerve, are a heterogeneous population of neurons with more than 40 molecularly distinct subtypes in mouse. While most RGCs, including the ON-OFF direction-selective RGCs (ooDSGCs), are vulnerable to axonal injury, a small population of RGCs, including the intrinsically photosensitive RGCs (ipRGCs), are more resilient.

**Results:**

By performing systematic analyses on RNA-sequencing data, here we identify lncRNAs that are expressed in ooDSGCs and ipRGCs with and without axonal injury. Our results reveal a repertoire of different classes of lncRNAs, including long intergenic noncoding RNAs and antisense ncRNAs that are differentially expressed between these RGC types. Strikingly, we also found dozens of lncRNAs whose expressions are altered markedly in response to axonal injury, some of which are expressed exclusively in either one of the types. Moreover, analyses into these lncRNAs unraveled their neighboring coding genes, many of which encode transcription factors and signaling molecules, suggesting that these lncRNAs may act *in cis* to regulate important biological processes in these neurons. Lastly, guilt-by-association analysis showed that lncRNAs are correlated with apoptosis associated genes, suggesting potential roles for these lncRNAs in RGC survival.

**Conclusions:**

Overall, the results of this study reveal RGC type-specific expression of lncRNAs and provide a foundation for future investigation of the function of lncRNAs in regulating neuronal type specification and survival.

**Supplementary Information:**

The online version contains supplementary material available at 10.1186/s12864-021-08050-x.

## Background

Long noncoding RNAs (lncRNA) have been shown play vital roles in regulating gene expression networks in developmental, physiological, and pathological processes. LncRNAs are a diverse class of transcribed RNAs, defined as transcripts with lengths exceeding 200 nucleotides that do not encode proteins. Broadly, lncRNAs are classified into four types: i) long intergenic noncoding RNAs (lincRNA) transcribed from intergenic regions; ii) intronic lncRNAs, transcribed entirely from introns of protein-coding genes; iii) sense lncRNAs, transcribed from the sense strand of protein-coding genes, and iv) antisense lncRNAs (AS lncRNAs), transcribed from the antisense strand of protein-coding genes. The majority (~ 78%) of lncRNAs are exemplified as tissue-specific, as opposed to only ~ 19% of mRNAs [1]. In addition, lncRNAs are characterized by higher developmental stage- and cell type-specificity in the central nervous system (CNS) than the mRNA counterparts [2].

Retinal ganglion cells (RGCs), which are the sole output neurons in the retina, send projections to the brain, and convey visual information. During the early developmental stage, combinations of transcription factors determine the fate of RGCs [3–6]. Remarkably, despite general features, RGCs also acquire subtype-specific characteristics that are strictly related to particular functions. In fact, there are more than 40 different subtypes of RGCs in the mouse, most of which are molecularly and physiologically distinct from each other [7–10]. However, the molecular mechanisms by which different RGC subtypes are specified during development remain unclear.

Another prominent feature of RGCs is the type-specific differences in their response to an injury. Studies have demonstrated that several subclasses of alpha RGCs and intrinsically photosensitive RGCs (ipRGCs) are particularly resilient to various types of insults, including axonal injury, whereas many direction selective-RGCs (DSGCs) are vulnerable to axonal injury [11–16]. Although several studies have described distinct transcription factors and signaling molecules that might contribute to the differential responses of injured RGCs, our understanding of the underlying mechanisms remain fragmentary.

IpRGCs are a group of neurons that express the photopigment melanopsin [17–19]. They have unique molecular and functional features quite distinct from other RGCs. In mice, although ipRGCs are generated as early as embryonic day 1, they follow a delayed developmental time course relative to other RGCs. ipRGC neurogenesis extends beyond that of other RGCs, and ipRGCs begin innervating their targets at postnatal ages, unlike most RGCs, which innervate their targets embryonically [20].

DSGCs are a major RGC type that represent approximately 20% of the total RGC population [21, 22]. DSGCs are broadly grouped based on two criteria. First, they either respond to both light onset and offset (ON-OFF) or just to the former (ON). Second, they prefer different directions of motion, giving rise to four types of ON-OFF DSGCs and three types of ON DSGCs. The ooDSGCs detect motion in one of four cardinal axes dorsal, ventral, temporal, and nasal directions [23, 24]. Previous studies have also shown that the ventrally tuned (VT) ooDSGCs (hereafter referred as ooDSGCs unless specified) are fluorescently tagged with reporter in the HB9-GFP BAC transgenic mice. Using this mouse line, it was shown that these ooDSGCs are particularly vulnerable to axotomy [13].

To investigate the molecular mechanisms underlying the exceptional survival and regenerative ability of ipRGCs after axonal injury, we previously performed RNA-sequencing (RNA-seq) on these RGCs and the ooDSGCs [11]. Subsequently, we had reported hundreds of coding genes that were uniquely expressed in these RGC types with and without optic nerve crush injury. In addition to our study, others have reported expression profiles of coding genes that were differentially expressed between various RGC types [9, 10]. However, RGC type-specific expression of lncRNAs has not been systematically examined. In this study, we analyze RNA-seq data from purified murine RGCs and identify lncRNAs that are expressed in two contrasting RGC types (i.e. injury-resilient ipRGCs and the injury-vulnerable ooDSGCs) in normal and optic nerve-injured animals.

## Methods

### Identification of lncRNAs by analyzing the RNA-seq data

RNA-seq data obtained from isolated ipRGCs and ooDSGCs of optic nerve axotomized (i.e. 3 days post crush) and normal (i.e. sham surgery) mice [11] were used for the lncRNA analyses. RNA-seq data were previously deposited under the accession number GEO: GSE115661 [11]. For the analyses in this current study, trimmed reads were aligned to the mm10 version of the mouse genome with STAR [25], followed by an assembly using the StringieTie2 [26]. For the alignment and assembly, we used GTF file from GENCODE version M17 (vM17; release 04/2018).

To classify the transcripts as putative novel lincRNAs, we used the method described in [27] with some modifications. Briefly, all reads that overlapped or were in a window of ±2 kb of the known mouse GENCODE genes were removed. Next, we generated transcriptome assemblies using StringTie2 for each of these samples separately and then used Cuffmerge to combine all annotations. All transcripts that were identified in these analyses as class code ‘u’ by Cuffmerge (class code “u” – putative novel intergenic transcripts) were retained. Transcripts with length < 200 nt and/or monoexonic transcripts were removed. Additionally, all transcripts with coding potential, as assessed by the Coding Potential Calculator (CPC version 2) [28] and CPAT [29] were discarded, resulting in a set of novel lincRNAs. This set was then combined with the mouse GENCODE vM17 transcripts, generating a list of lncRNAs (antisense lncRNAs and lincRNAs from GENCODE and novel lincRNAs identified in this work) that are expressed in ipRGCs and ooDSGCs.

### Differential expression analysis

The RNA abundance defined as the FPKM (Fragments Per Kilobase Million), TPM (Transcripts per Million), and the sum of exon read count per gene was calculated using RSEM [30], and differential expression (DE) analysis was performed with DESeq2 [31]. A gene was considered detected if the FPKM was > 0.3 in at least two replicates of one sample and significantly changed if the adjusted *p*-value was < 0.05.

### RGC type-specific expression

To evaluate RGC type specificity, we calculated the fractional expression level (FEL) [32] for each transcript and RGC sample, corresponding to the fraction of the cumulative TPM detected in all samples under study. We used a FEL threshold of greater than 50% to consider a transcript as displaying RGC type-specific expression.

### Gene ontology (GO) enrichment analysis

We used DAVID v6.8 to perform GO enrichment analysis [33]. “Biological Process” and “Molecular Function” GO terms with *p*-value < 0.05 were defined as enriched.

### Nearest-neighbor gene analysis of lncRNA

Protein-coding genes within 300 kb upstream or downstream of differentially expressed (DE) lncRNA locus were considered as lncRNA neighbors and its putative *cis*-targets. This analysis was performed separately for each dataset of DE lncRNAs; injured ipRGCs vs. normal ipRGCs; injured ooDSGCs vs. normal ooDSGCs; injured ooDSGCs vs. injured ipRGCs; normal ooDSGCs vs. normal ipRGCs. Only the DE mRNAs were considered in the analysis. We calculated a Pearson correlation coefficient based on the TPM expression profiles for each lncRNA-nearby gene pair in the four RGC groups described in this study and in the major RGC types identified in a previous single cell RNA-seq study [9]. See the sub-section below for a more detailed description. LncRNA- nearby gene pairs with r > |0.5| were considered as correlated.

### Gene expression correlation analysis of lncRNA and mRNA

To predict the potential role of lncRNAs, we performed guilt-by-association analysis [34]. We constructed Pearson correlation matrices including the DE mRNAs and the DE lncRNA in the following RGC group comparisons: injured ipRGCs vs. normal ipRGCs; injured ooDSGCs vs. normal ooDSGCs; injured ooDSGCs vs. injured ipRGCs; and normal ooDSGCs vs. normal ipRGCs. Pearson correlation values of TPM expression profiles were calculated for each lncRNA to all mRNAs across the four RGC groups from our study as well as the uninjured control and 4 days post-crush expression data from a previous work that had identified RGC types using single cell RNA-seq (scRNAseq) [9]. We re-analyzed the RGC scRNAseq data using the same gene annotation used in our RGC data in order to detect the reference lncRNAs (GENCODE version M17) and the novel lncRNAs reported in our work. As we have done for ipRGCs and ooDSGCs, which we categorize as two major RGC types without further dividing them into individual RGC subtypes (i.e. M1-M6 ipRGCs), we performed a supervised clustering using the RGC markers described in Tran et al. study [9] to group RGCs into 6 major types: ooDSGCs, ipRGCs, alpha-RGCs, Tusc5/W3/F-RGCs, T-RGCs, and N-RGCs. The Seurat pipeline was utilized to cluster the cells employing the resolution = 1.8.

For each lncRNA, we considered its correlated protein-coding mRNAs, transcripts with a cutoff at Pearson correlation coefficient of − 0.88 > *r* > 0.88. The protein-coding genes were grouped in correlation clusters (rows), based on their positive or negative correlation with the lncRNAs. The protein-coding genes clusters were manually selected using the hierarchical clustering dendrogram. Gene Ontology enrichment analysis of mRNAs correlated with lncRNAs was performed separately for each DE comparison. Since we found only a few correlated lncRNA- mRNA pairs in the injured ipRGCs vs. normal ipRGCs comparison, we did not perform such analysis for this RGC group comparison. For each DE comparison, we used the mRNAs in the lncRNA-mRNA correlation clusters for performing the GO analysis (see above).

### Animals

C57BL6/J and B6.Cg-Tg(Thy1-CFP)23Jrs/J (hereafter referred as Thy1-CFP] mice between 9 and 11 weeks of age were used in this study. All experimental procedures were performed in compliance with protocols approved by the Institutional Animal Care and Use Committee (IACUC) at University of Miami (Permit Number: 19–150). For optic nerve crush, mice were anaesthetized with isoflurane, and buprenorphine (0.05 mg/kg) was administrated as post-operative analgesic. This study was carried out in compliance with the Animal Research: Reporting of In Vivo Experiments (ARRIVE) guidelines.

### Optic nerve crush

For the injury procedure, the left optic nerve was exposed intraorbitally and crushed with jeweler’s forceps (Dumont #5, Roboz) for 10 s approximately 1 mm behind the optic disc.

### Tissue preparation and immunohistochemistry

Thy1-CFP mice were perfused transcardially with PBS followed by 4% paraformaldehyde (PFA) in PBS, then eyes were dissected and postfixed with 4% PFA in PBS overnight at 4 °C. Samples were cryoprotected by incubating in 30% sucrose in PBS for 48 h. Eyes were cryosectioned to 16-μm thickness. Tissue sections were blocked in 5% normal donkey serum and 0.3% Triton X-100 in PBS for 1 h and incubated with primary antibodies diluted in blocking buffer overnight at 4 °C, followed by 1-h incubation with secondary antibodies at room temperature. Primary antibodies used were rabbit anti-RBPMS 1:200 (PhosphoSolutions; cat# 1830) and chicken anti-GFP 1:1000 (Abcam; cat#13970).

### Retinal ganglion cell purification

Three days after optic nerve crush, retinas were dissociated using papain digestion (17 U/ ml papain, Worthington; 5.5 mM L-cysteine; 0.006% DNase; 1.1 mM EDTA in DMEM/2% B27) during 30 min at 37 °C as previously described at [35]. After digestion, retinas were washed in DMEM, gently triturated using a Pasteur pipet and centrifuged at 300 g × 5 min at RT. Dissociated cells were resuspended in DMEM/2% B27, passed through a 35 μm cell strainer and placed on ice until use. Dissociated retinal cells were stained with Ghost red 780 (TONBO Biosciences) to exclude non-viable cells and then CFP cells were separated by BD FACS SORP Aria-IIu (BD Biosciences) at Flow Cytometry Shared Resource, University of Miami, Sylvester Comprehensive Cancer Center. C57BL6/J retinal cells were used as unstained control. Sorted cells were collected in PBS and immediately frozen at − 80 °C.

### Reverse transcription and quantitative real-time PCR (RT-qPCR)

We validated the expression of one randomly selected novel lincRNA [*XLOC_020964*] in purified RGCs using RT-PCR. PCR product was analyzed by Sanger sequencing at GENEWIZ. The primers used are listed in Additional file 1: Table S1. To validate RGC purification (Fig. S4B and C), total RNA of FACS isolated RGCs from Thy1-CFP mice was extracted using RNeasy Micro Kit (Qiagen) and treated with DNase I according to the manufacturer’s instructions. 500 pg of total RNA was poly(A) amplified and reverse transcribed with MessageBOOSTER™ cDNA Synthesis Kit for qPCR (Lucigen). Quantitative real-time PCR was performed to measure specific genes used as controls [*Thy1* and *Slc17a6* as markers for RGCs, and *Rho* as a marker for rods) using PowerUp™ SYBR® Green Master Mix (Applied Biosystems) on a QuantStudio 3 (Applied Biosystems). *Hprt* (hypoxanthine guanine phosphoribosyl transferase) was used as the endogenous control.

### RNA-fluorescent in situ hybridization (RNA-FISH)

RNA-FISH was performed using the RNAscope kit (RNAscope® Multiplex Fluorescent Reagent Kit v2; Catalog No. 323100) according to the manufacture’s protocol (ACD-Bio). Target probes used are as follows: RNAscope® Probe- Mm-melanopsin [*Opn4*] (Cat No. 438061), RNAscope® Probe- Mm- cocaine- and amphetamine-regulated transcript protein [*Cartpt*]-C2 (Cat No. 432001-C2], RNAscope® Probe- Mm-Cartpt (Cat No. 432001), RNAscope® Probe - Mm-Ecel1-C2 (Cat No. 475331-C2], RNAscope® Probe - Mm-Gm29374–201-C3 (Cat No. 1073601-C3] for lncRNA *RP23-416O18.4* and RNAscope® Probe - Mm-TCONS-00067968-C3 (Cat No. 1041891-C3] for lncRNA *XLOC_020964*. TSA-based fluorophores were from Perkin Elmer (TSA Plus Fluorescein, PN NEL741001KT; TSA Plus Cyanine 3, PN NEL744001KT; TSA Plus Cyanine 5, NEL745001KT). Imaging was done using a Nikon Ti epifluorescence microscope.

### RNA-FISH lncRNA expression quantification

To quantify lncRNA expression, we evaluated the number of RNA speckles detected in ooDSGCs and ipRGCs of injured and sham control retinal sections from C57BL6/J mice. For quantifying *XLOC_020964* expression in ooDSGCs and ipRGCs, fluorescent speckles detected in individual *Cartpt*-positive and *Opn4*-positive cell in the ganglion cell layer were evaluated. A cell was considered positive for the presence of the lncRNA if a minimum of four fluorescent speckles were detected. A total of 163 *Cartpt*-positive cells from 2 injured mice and 309 *Cartpt*-positive cells from 2 sham control mice, and 41 *Opn4*-positive cells from 2 injured mice and 77 *Opn4*-positive cells from 2 sham control mice were analyzed. A total of 20–28 sections from 2 animals per group were analyzed. For quantifying *RP23-416O18.4* and *Ecel1* co-expression in ooDSGCs and ipRGCs, fluorescent speckles detected in individual *Cartpt*-positive and *Opn4*-positive cells in the ganglion cell layer were evaluated. A total of 136 *Cartpt*-positive cells from 2 injured mice and 259 *Cartpt*-positive cells from 2 sham control mice, and 37 *Opn4*-positive cells from 2 injured mice and 84 *Opn4*-positive cells from 2 sham control mice were analyzed. A total of 20–28 sections from 2 animals per group were analyzed.

### Statistical analysis

RNAseq differential expression analysis was performed using DESeq2 [31]. Wald test *p*-values adjusted for multiple testing using the procedure of Benjamini and Hochberg were used.

Values of adjusted p-value < 0.05 were considered significant. DAVID was used for gene ontology enrichment analysis and the EASE Score/ *p*-value < 0.05 [33] was considered significant. For RGC type-specific expression, we used the chi-square test to compare the FEL for lncRNAs and mRNAs. In the gene expression correlation analysis, lncRNA-mRNA pairs with Pearson correlation coefficient (r) > |0.5|, p-value < 0.05 (lncRNA-nearby mRNAs) or r > |0.88|, p-value < 0.05 were considered significantly correlated. Two sample t-test, one tail was used to evaluate significance for RNA-FISH quantification.

## Results

### Identification of lncRNAs in ipRGCs and ooDSGCs

To identify the repertoire of lncRNAs that are expressed in ipRGCs and ooDSGCs, we analyzed the RNA-seq data obtained from isolated murine ipRGCs and ooDSGCs [11]. In addition to the normal uninjured condition, we examined lncRNA expression of these RGC types extracted 3 days after intraorbital optic nerve crush [11]. Thus, we analyzed a total of four RGC groups: normal ipRGCs, injured ipRGCs, normal ooDSGCs and injured ooDSGCs.

**Fig. 1:**
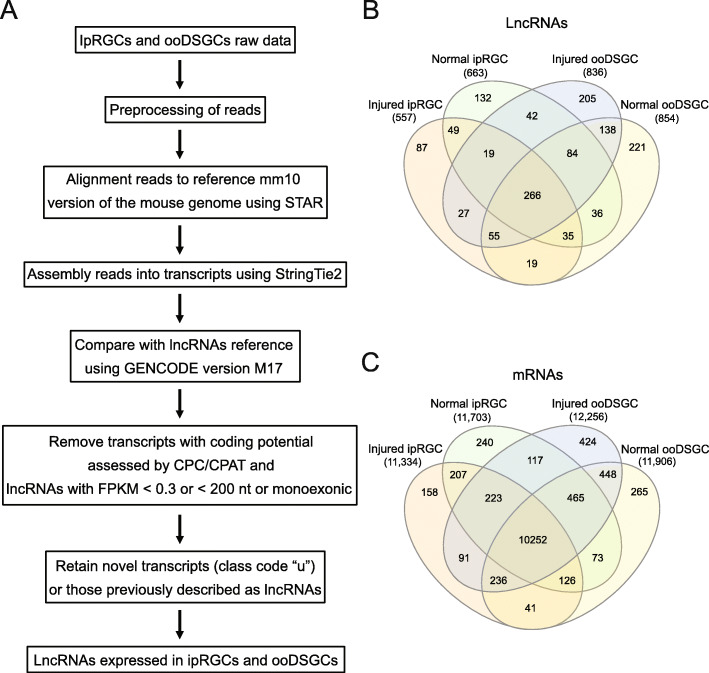
LncRNA transcriptome of ipRGCs and ooDSGCs. **a** Schematic of procedure used for lncRNA identification. Venn diagram showing the number of lncRNAs (**b**) and protein-coding mRNAs (**c**) expressed in ipRGCs and ooDSGCs under normal and injury (i.e. optic nerve crush) conditions

RNA-seq reads were aligned to the genome and assembled using a reference transcriptome that includes the lncRNAs from the mouse GENCODE vM17 with novel lncRNAs identified in this work (Fig. 1a). This generated a catalog of the lncRNAs, including antisense lncRNAs and lincRNAs that are expressed in ipRGCs and ooDSGCs (Additional file 2: Table S2).

Overall, we detected a total of 1415 lncRNAs (Fig. 1b), of which 347 (25%) were identified as putative novel lincRNAs (i.e. not present in the GENCODE vM17) expressed in at least one of the four RGC groups (Additional file 9: Fig. S1c). Comparing lncRNA and protein-coding mRNA expression profiles we found that 31% (268/851) and 49% (564/1147) of the lncRNAs were detected exclusively in ipRGCs and ooDSGCs (Fig. 1b and Additional file 3: Table S3), respectively. In contrast, for the mRNAs, only 5% (605/12,229) and 9% (1137/12,761) were detected exclusively in ipRGCs and ooDSGCs, respectively (Fig. 1c and Additional file 3: Table S3). These data suggest a higher RGC type-specific expression for lncRNAs compared to mRNAs (chi-square test; *p* < 0.0001). We used a Fractional Expression Level (FEL) [32] threshold to compare the lncRNA and mRNA expression patterns across the four RGC types. Using a FEL threshold of greater than 50% to consider a transcript as displaying high specificity for RGC types, we observed that 56% (797/1415) of the lncRNAs show RGC type-specific profiles, compared to a smaller fraction for the mRNAs (19%; 2517/13,366) (chi-square test; p < 0.0001) (Additional file 9: Figs. S2a-b and S2d). To discard the possibility that the high specificity of lncRNAs is due to lncRNAs’ low expression levels, we also calculated the FEL for lncRNAs and mRNAs detected at similar expression levels (Additional file 9: Figs. S2c and S2d). For this we used the lncRNAs [*n* = 478) and mRNAs [*n* = 3735) that have a maximal expression level within the range of 4–20 TPM (Additional file 9: Fig. S2c). We observed similar results compared to the analysis using all transcripts in the full TPM range (Additional file 9: S2d); 53% (252/478) of the lncRNAs presented FEL greater than 50%, while only 27% (1007/3735) presented this pattern for the mRNAs (chi-square test; *p* < 0.0001) (Additional file 9: Fig. S2d). Thus, in agreement with previous reports [1], our data show that lncRNAs exhibit higher RGC-type specificity than protein-coding genes at different expression ranges.

We examined injury-specific lncRNAs. Like the RGC type specificity, our data show that higher numbers of lncRNAs are expressed exclusively in either normal or injury condition than the protein-coding genes (Fig. 1b, c and Additional file 3: Table S3).

Next, we compared our lincRNA data, including novel lincRNAs, to the lincRNA data from whole retina RNA-seq [36]. Reflecting the fact that ipRGCs and ooDSGCs represent only a small fraction of the total retinal cells [8], we found that only 15% (58/388) of lincRNAs, including novel lincRNAs, identified in normal ipRGCs were also identified in the whole retina RNA-seq [36] (Additional file 4: Table S4). For normal ooDSGCs, we found that 12% (61/511) of lincRNAs were also seen in the whole retina RNA-seq (Additional file 4: Table S4). Of the lincRNAs seen in the whole retina RNA-seq (272 transcripts, with a minimum FPKM > 0.3), 27% (74/272) were also detected in at least one of the two RGC types. Among these 74 transcripts, 61% (45/74) were detected in both RGC types, suggesting they may represent broadly expressed lncRNAs.

### General characteristics of lncRNAs expressed in ipRGCs and ooDSGCs

**Fig. 2:**
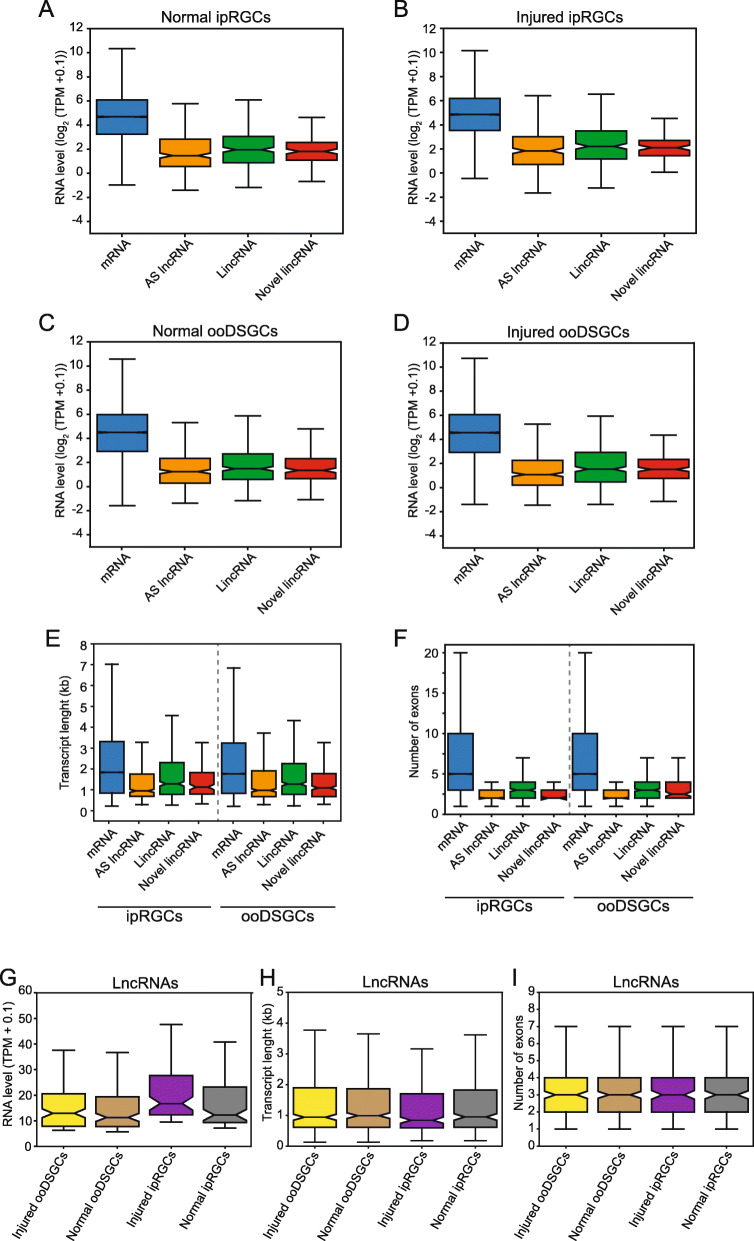
Expression level and genomic features of the lncRNAs detected in ipRGCs and ooDSGCs. Abundance of lncRNAs compared to the protein-coding mRNAs detected in ipRGCs (**a** and **b**) and ooDSGCs (**c** and **d**) under injury and normal conditions. Comparative analysis of the transcript length (**e**) and number of exons (**f**) of the lncRNAs and protein-coding mRNAs detected in ipRGCs and ooDSGCs. Abundance (**g**), transcript length (**h**) and number of exons (**i**) for the top 25% most highly expressed lncRNAs detected in ipRGCs and ooDSGCs under normal and injury conditions. AS, antisense; lincRNA, long intergenic noncoding

To further examine lncRNAs transcribed in ipRGCs and ooDSGCs, we subdivided them according to their genomic location in relation to the protein coding genes. Antisense lncRNAs are transcribed from the opposite strand of the protein coding genes, whereas lincRNAs are located between protein coding genes. The numbers of antisense lncRNAs, lincRNAs, and novel lincRNAs detected for each condition are shown in Additional file 2: Table S2 and Additional file 9: Fig. S1. In line with the previous studies [1, 37], we observed that lncRNAs have lower abundance compared to protein-coding genes (Fig. 2a-d) (t-test; *p*-value < 0.001).

Additionally, we compared the abundance of the lncRNAs detected in RGCs with the abundance of the lincRNAs detected in the whole retina RNA-seq (Additional file 9: Figs. S3a and S3b) [36]. However, we did not find a statistically significant difference in this comparison.

We found that, on average, lncRNAs were shorter than mRNAs in length (t-test, *p*-value < 0.001; Fig. 2e). Moreover, lncRNAs were less spliced than protein-coding genes (t-test, p-value < 0.001; Fig. 2f). Figure 2 shows the abundance (Fig. 2g), transcript length (Fig. 2h) and number of exons (Fig. 2i) for the top 25% most highly expressed lncRNAs detected in ipRGCs and ooDSGCs under normal and injury conditions. Compared to the whole retina lincRNAs, the average length of RGC lncRNAs was shorter (t-test, p-value < 0.001; Additional file 9: Fig. S3c). Similarly, the average number of exons in RGCs was smaller compared to that of the whole retina lincRNAs (t-test, p-value < 0.001; Additional file 9: Fig. S3d).

### LncRNAs differentially expressed in ipRGCs and ooDSGCs

**Fig. 3:**
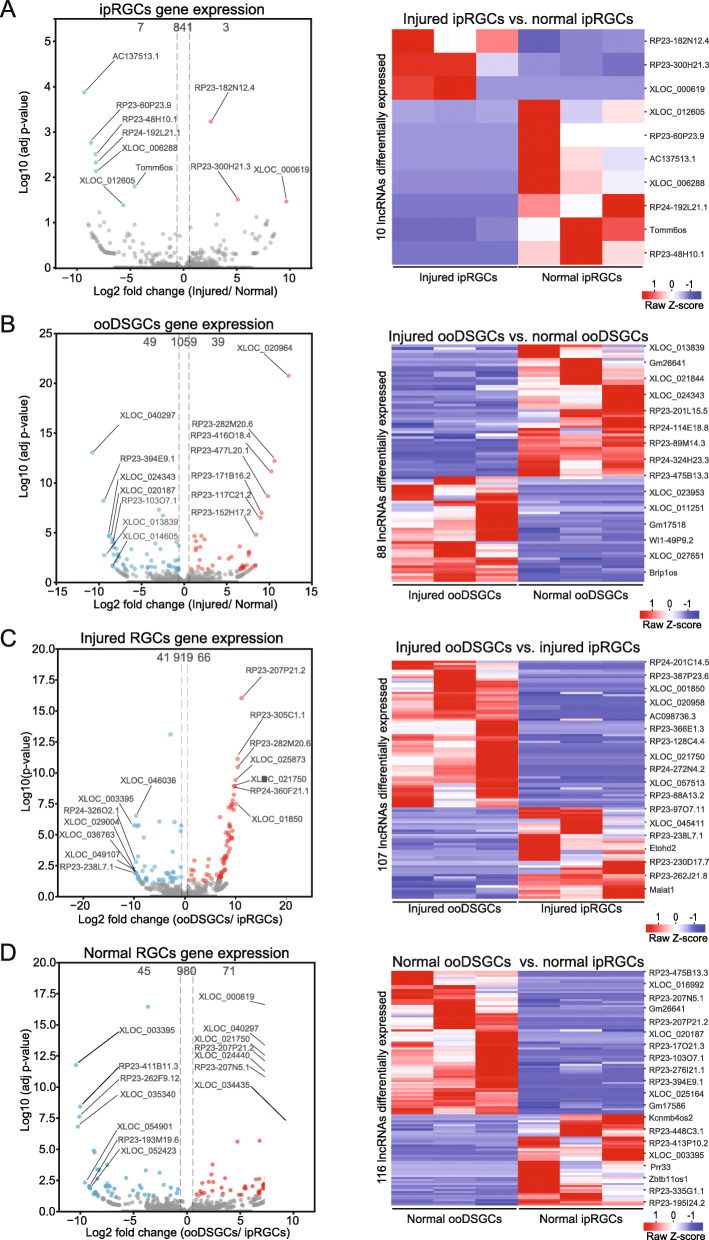
Volcano plots and heat maps showing the level of gene expression for the lncRNAs differentially expressed in the two RGC types under injury and normal conditions. **a** Injured ipRGCs vs. normal ipRGCs. **b** Injured ooDSGCs vs. normal ooDSGCs. (**c**) Injured ooDSGCs vs. injured ipRGCs. **d** Normal ooDSGCs vs. normal ipRGCs. Red and blue dots indicate differentially expressed genes; gray dots indicate genes that are not differentially expressed. Expression values in the heat maps were based on Z-score normalized TPM for each lncRNA

Since very little is known about lncRNAs that are expressed in different RGC types, we sought to identify differentially expressed lncRNAs. We detected 10 lncRNAs that were differentially expressed (adj p-value < 0.05) between normal and injured ipRGC groups, and 88 lncRNAs differentially expressed between normal and injured ooDSGC groups (Fig. 3a and b). Only 3 putative novel lincRNA were differentially expressed between normal and injured ipRGCs, whereas 27 putative novel lincRNAs were differentially expressed between normal and injured ooDSGCs. Thus, although the amount of lncRNAs expressed in these RGC types is similar (851 transcripts in ipRGCs and 1147 in ooDSGCs; Fig. 1b), the number of lncRNAs whose expression is significantly altered by injury is markedly lower in ipRGCs compared to ooDSGCs (Fig. 3a and b).

We sought to identify lncRNAs that are differentially expressed between injured ooDSGCs and injured ipRGCs as well as those that are differentially expressed between normal ooDSGCs and normal ipRGCs. For the injury group comparison, we detected 107 lncRNAs that were differentially expressed, whereas for the normal group comparison, we identified 116 differentially expressed lncRNAs (Fig. 3c and d). Of these lncRNAs, 28 and 39 correspond to putative novel lincRNAs for each comparison, respectively. A full list of the differentially expressed lncRNAs is provided in Additional file 5: Table S5.

**Fig. 4:**
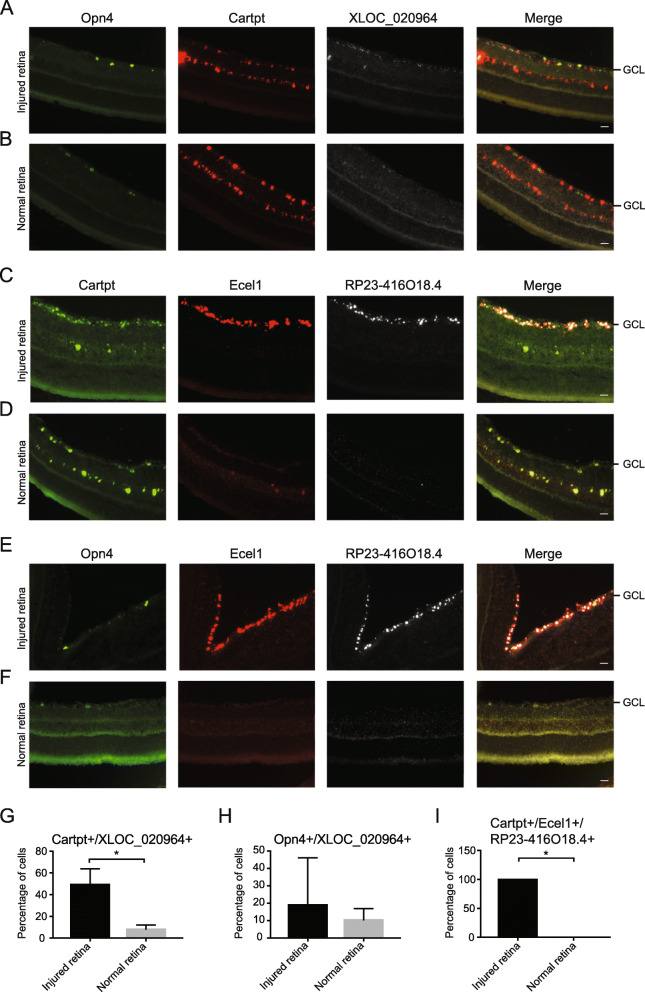
Fluorescent in situ hybridization validation of two lncRNAs upregulated in injured ooDSGCs. Retina sections 3 days post crush (injured) (**a**) and sham control (normal) (**b**), probed for *Opn4* (green), *Cartpt* (red) and *XLOC_020964* (white). Injured (**c, e**) and normal retina sections (**d, f**), probed for *Cartp*t (green; **c, d**) or *Opn4* (green; **e**, **f**), *Ecel1* (red) and *RP23-416O18.4* (white). *Ecel1* is the closest neighbor of the lincRNA *RP23-416O18.4* and both are differentially upregulated in injured ooDSGCs. Quantification of the percentage of ooDSGCs (**g**) and ipRGCs (**h**) cells expressing the novel lncRNA *XLOC_020964* in retina sections 3 days post crush and sham control. Values were normalized to the total of Cartpt+ or Opn4+ cells. (**i**) Quantification of the percentage of ooDSGCs cells co-expressing the *lincRNA RP23-416O18.4* and *Ecel1* in retina sections 3 days post crush and sham control. Values were normalized to the total of Cartpt+/Ecel1+ cells. * T-test, *p* < 0.05. Scale bar, 25 μm. Ganglion cell layer (GCL)

Next, we validated the differential expression of one novel lncRNA, *XLOC_020964* using RNA-fluorescent in situ hybridization (RNA-FISH). From our RNA-seq analysis, we found that this lncRNA is highly upregulated in the injured ooDSGCs compared to the normal ooDSGCs: TPM value for the injured ooDSGCs was 41 and for the normal ooDSGCs it was 0. *XLOC_020964* was not detected in the injured and normal ipRGCs. Consistently, RNA-FISH results show obvious increase in *XLOC_020964* expression in the ooDSGCs after injury (Fig. 4a-b, g-h]. Additionally, we validated the expression of this novel lincRNA using RT-PCR (Additional file 9: Fig. S4).

### Functional annotation of lncRNA-nearby protein-coding mRNA

Several studies have shown that lncRNAs can regulate their neighboring genes in a *cis*-acting manner. These *cis*-acting lncRNAs constitute a sizeable fraction of lncRNAs, and regulate gene expression in a manner dependent on the location of their own sites of transcription, at varying distances from their targets [38]. To probe the potential roles of differentially expressed lncRNAs, we examined the differentially-expressed protein-coding genes transcribed within 300 kb (upstream or downstream) of lncRNA *loci*. Using this criterion, we identified 2 nearby protein-coding genes in injured ipRGCs vs. normal ipRGCs (comparison 1), 28 in injured ooDSGCs vs. normal ooDSGCs comparison (comparison 2), 37 in injured ooDSGCs vs. injured ipRGCs (comparison 3) and 43 in normal ooDSGCs vs. normal ipRGCs (comparison 4). Next, we calculated Pearson correlation coefficient based on the TPM expression profiles for each lncRNA- nearby gene pair in the four RGC groups described in this study. To expand our analysis, we sought to incorporate data from broader RGC types. To this end, we added to Pearson correlation analysis the TPM expression profiles for each lncRNA- nearby gene pair from the major RGC types identified previously in a single cell RNA-seq study [9] (see Methods section). Accordingly, we found a group of lncRNAs positively or negatively correlated with their nearby protein-coding genes (r > |0.5|) (i.e. 0, 12 (43%), 12 (32%) and 22 (51%) in comparisons 1, 2, 3 and 4 (listed above), respectively) (Additional file 9: Figs. S5 and S6; Additional file 6: Table S6).

Notably, among the correlated neighboring genes, we found protein-coding genes whose protein products are known to regulate cell death and apoptosis. For example, nearest-neighbor analysis identified *Ecel1*, a known injury-induced gene, as the closest neighbor of the lincRNA *RP23-416O18.4* (Gm29374) (r = 0.72) (Additional file 6: Table S6). Our data showed that this lncRNA is one of the most highly upregulated lncRNAs in ooDSGCs after axonal injury. Similarly, our RNA-seq showed that *Ecel1* mRNA expression is highly induced in ooDSGCs after axonal injury, to an extent far greater than the level seen in ipRGCs; *Ecel1* expression is approximately 11-fold higher for injured ooDSGCs compared to injured ipRGCs. *Ecel1* was not detected in normal ooDSGCs and in normal ipRGCs [11]. We confirmed induction and co-expression of *RP23-416O18.4* and *Ecel1* in injured ooDSGCs using RNA-FISH (Fig. 4c-f and i). In contrast, as shown in Fig. 4, we did not detect *Ecel1* and lincRNA *RP23-416O18.4* in ipRGCs.

Notably, among the nearby protein-coding genes that were correlated with lncRNAs differentially expressed between normal ooDSGCs and normal ipRGCs, there were transcription factors known to be expressed specifically in these two RGC types. For example, *Eomes* (also known as *Tbr2*] was identified in our analysis as a neighbor of a lncRNA detected exclusively in ipRGCs (r = 0.65) (Additional file 6: Table S6). *Eomes* knockout was shown previously to cause death of ipRGCs in uninjured mice [4] as well as in mice with optic nerve crush [11], demonstrating that *Eomes* is essential for the survival of ipRGCs.

Additionally, we identified *Pou4f1* (also known as *Brn3a*] and *Pou4f3* (also known as *Brn3c*] as neighbors of lncRNAs that were detected exclusively in ooDSGCs (r = 0.74 and r = 0.71 for *Pou4f1* and r = 0.65 for *Pou4f3*] (Additional file 6: Table S6). The Brn3/Pou4f transcription factors are known to participate in RGC development and type specification [5, 39]. We and others have previously shown that ooDSGCs, but not ipRGCs, exhibit high levels of *Brn3a* and *Brn3c* expression [10, 11, 40], raising the possibility that these lncRNAs may play functional roles in regulating expression of these genes and promoting RGC type specification.

### Functional annotation of gene-lncRNA co-expression networks

**Fig. 5:**
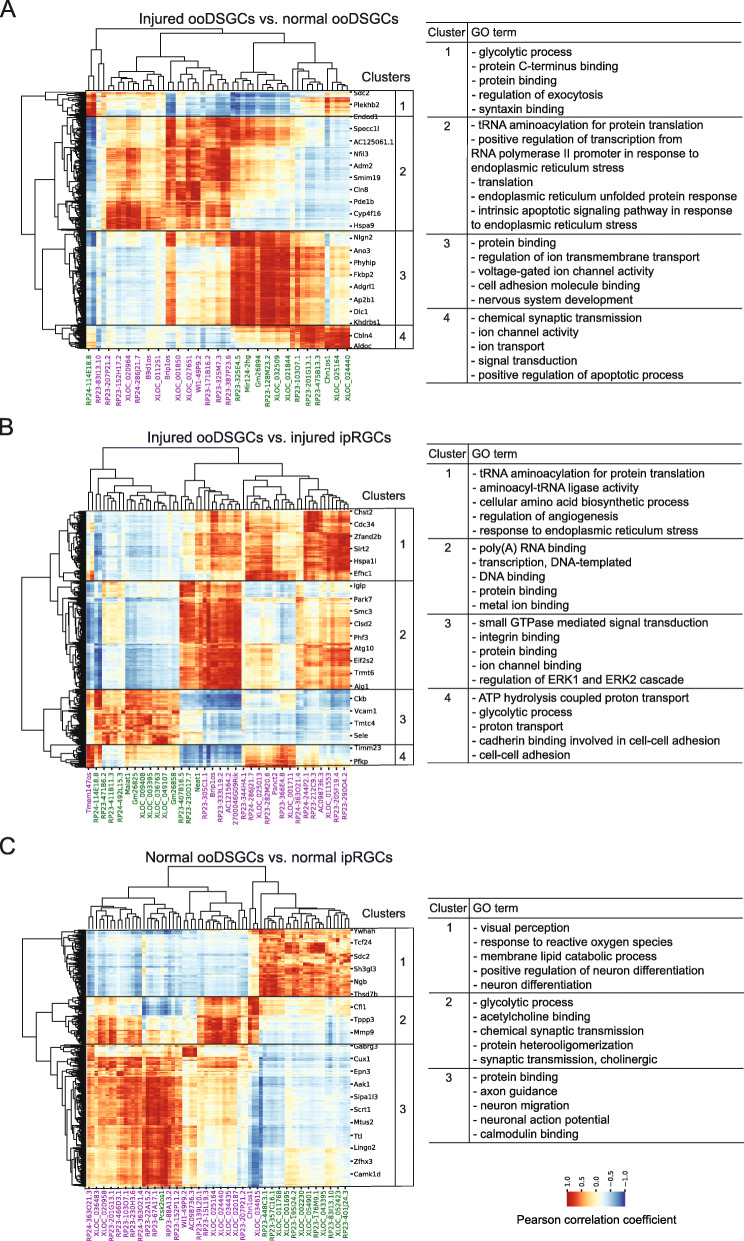
Biological associations arising from the lncRNAs correlated with protein-coding genes in RGCs. Heat map with correlated lncRNAs (columns) and mRNA (rows) (r > 0.88 or r < − 0.88) are shown for each group of DE mRNAs and DE lncRNAs in the RGC group comparisons: injured ooDSGCs vs. normal ooDSGCs (**a**), injured ooDSGCs vs. injured ipRGCs (**b**) and normal ooDSGCs vs. normal ipRGCs (**c**). The correlation between DE lncRNAs and DE mRNAs was calculated based on the expression profiles from ipRGCs and ooDSGCs of our study and from the major RGC types identified in the single cell RNA-seq study [9]. In each group comparison, the DE mRNAs were grouped in correlation clusters and used for Gene Ontology enrichment analysis (GO). Five terms among the top ten GO enriched terms in each cluster are shown on the right of each panel. Red to blue Pearson Correlation scale indicates the degree to which mRNA expression is positively (dark red), negatively (dark blue), or not correlated with the expression of the respective lncRNA. LncRNAs in purple and green fonts are upregulated and downregulated, respectively, in each RGC group comparison

LncRNAs can regulate gene transcription in *cis* and *tran*s, where trans-acting lncRNAs exert their function at a different location to which they are transcribed. Therefore, we performed guilt-by-association analysis [34] to predict the putative roles of lncRNAs seen in our study. Pearson correlation coefficient was calculated using the similar approach described above. The difference for this analysis was that we used the whole set of differentially expressed lncRNAs and differentially expressed mRNAs, but excluded the 300 kb nearby requirement. In this analysis, we identified potential associations based on Pearson correlated coefficient (r > |0.88|) for the differentially expressed lncRNAs and mRNAs in each RGC group comparison (Fig. 5). Each lncRNA shown in Fig. 5 was correlated with at least one protein-coding gene (Additional file 7: Table S7). The protein-coding genes were grouped in correlation clusters, based on their positive or negative correlation with the lncRNAs. We manually selected the clusters of protein-coding genes (rows) in the Fig. 5 using the hierarchical clustering dendrogram. Then, we used GO enrichment analysis to infer functions of the lncRNAs correlated with mRNAs within each specific correlation cluster (Fig. 5, Additional file 8: Table S8).

We found 2 lncRNAs correlated to 3 mRNAs in the set of differentially expressed transcripts in normal ipRGCs vs. injured ipRGCs group comparison (Additional file 7: Table S7). Notably, one of the correlated mRNAs is ATF5, a gene known to be associated with early injury response and cell death [41].

For the set of differentially expressed transcripts in the normal ooDSGCs vs. injured ooDSGC comparison, the co-expressing pairs comprised 53 lncRNAs whose expression was correlated with a total of 717 mRNAs (Fig. 5a, Additional file 7: Table S7), and these mRNAs were grouped into four clusters. Functional analysis of the mRNAs in each cluster revealed enrichment of several GO “Biological Processes” and “Molecular Functions” terms including “glycolytic process”, “positive regulation of transcription from RNA polymerase II promoter in response to endoplasmic reticulum stress”, “intrinsic apoptotic signaling pathway in response to endoplasmic reticulum stress”, “cell adhesion molecule binding” and “nervous system development” (Fig. 5a, Additional file 8: Table S8). Fig. S7 (Additional file 9: Fig. S7) shows sub-groups of protein-coding genes from cluster 2 (e.g. *Chac1, Ddit3, Trib3 and Trp53*] that are associated with regulating RGC death after injury [41, 42].

Co-expression analysis for the injured ooDSGC vs. injured ipRGC comparison shows that 535 mRNAs were correlated with 68 lncRNAs and grouped in four clusters (Fig. 5b, Additional file 7: Table S7). The mRNAs in these clusters were significantly enriched in GO terms such as “response to endoplasmic reticulum stress”, “transcription, DNA-templated”, “protein binding”, “regulation of ERK1 and ERK2 cascade”, “glycolytic process” and “cell-cell adhesion” (Fig. 5b, Additional file 8: Table S8).

Lastly, for the normal ooDSGCs vs. normal ipRGCs comparison, 72 lncRNAs correlated with a total of 440 mRNAs, and these mRNAs were grouped in three clusters (Fig. 5c, Additional file 7: Table S7). The protein-coding genes in these clusters were significantly enriched in GO terms such as “visual perception”, “neuron differentiation”, “chemical synaptic transmission” and “neuron migration” (Fig. 5c, Additional file 8: Table S8).

## Discussion

LncRNAs are emerging as major controllers of gene expression networks in developmental, physiological, and pathological processes. Many lncRNAs show tissue- and cell-specific expression, particularly in the nervous system. LncRNAs regulate the transcription of proximal and distal protein-coding genes in *cis* and in *trans*, respectively. LncRNAs can either activate or repress protein-coding genes through different mechanisms [38, 43]. For example, lncRNAs can trigger gene transcription by recruiting chromatin activation complex such as TrxG/MLL, leading to deposition of histone 3 lysine 4 trimethylation (H3K4me3) at the gene promoters. Alternatively, lncRNAs can regulate nuclear positioning of enhancer, potentiating the enhancer to induce expression of the target genes. Others have shown that distinct lncRNAs interact with the chromatin remodeler PRC2 complex, resulting in methylation of histone H3 at lysine 27 (H3K27me3) and repression of gene transcription [44–47].

However, despite the recognition of lncRNAs as a major regulator of gene expression during development and in pathological conditions, the extent to which lncRNAs regulate RGC development and survival remains largely unknown.

We identified numerous lncRNAs that were differentially expressed between normal ipRGCs and normal ooDSGCs. As mentioned above, some of these lncRNAs have their expression correlated with nearby protein-coding genes that encode RGC type-specific transcription factors, including *Eomes, Brn3a,* and *Brn3c.* However, it remains unknown whether these differentially expressed lncRNAs in fact regulate the expression of these transcription factors and control RGC development.

Another notable finding in this study is the dozens of lncRNAs whose expressions were altered in response to axonal injury. Nearest-neighbor analysis identified apoptosis-related genes that are close neighbors of these lncRNAs. One example is the gene *Ecel1*, which has been studied extensively for its role in regulating RGC death and regeneration after injury [48–50]. Ecel1 protein, a membrane-bound metalloprotease, has been shown to prevent the activation of signaling pathways associated with apoptosis [48]. Correlated expression pattern of *RP23-416O18.4* and *Ecel1* seen in our nearest-neighbor analysis was similarly observed in two previous studies [9, 51]. A single cell RNA-seq performed on mouse RGCs has shown that *RP23-416O18.4* and *Ecel1* have the same expression pattern across different time-points after axonal injury [9]. In another study, RNA-seq was performed on whole retinas, and the results showed that these two genes are among the top 20 upregulated genes after optic nerve crush [51]. However, the role of *RP23-416O18.4* in RGC survival remains to be determined.

Interestingly, in our analysis, lncRNA *Neat1* was detected exclusively in the injured ipRGCs. A previous study using spinal cord neural progenitor cells showed that *Neat1* regulates neuronal differentiation, migration and apoptosis [52]. The authors also showed that overexpression of *Neat1* induces expression of Wnt/β-catenin signaling molecules including Wisp1, Wnt5a, and Wnt2. Moreover, overexpression of *Neat1* prevented apoptotic death of spinal cord neural progenitor cells in an Wnt/β-catenin dependent manner [52]. Since the Wnt/β-catenin pathway is known to promote RGC survival and axon regeneration [53], these observations raise a possibility that *Neat1* regulates the Wnt/β-catenin signaling pathway and contributes to promoting ipRGC axon regeneration and survival.

In our study, guilt-by-association analysis suggested that lncRNAs may be functionally correlated to protein coding genes enriched in specific “Biological Processes” and “Molecular Functions”. Notably, some lncRNAs were correlated with genes significantly enriched in “Biological Processes” related to apoptotic pathways (Fig. 5). Genes associated with RGC death, including *Chac1, Ddit3 and* Trib3 were previously shown to be highly expressed in injured ooDSGCs [11]. In our present study, we found that these genes were positively correlated with the lncRNAs *XLOC_020964*, *RP23-152H17.2*, *RP24-286 J21.7* and *XLOC_027651* (Additional file 7: Table S7).

Insulin growth factor 1 receptor [*Igf1r*] is a gene upregulated in the injured ipRGCs, that is essential to axonal regeneration in RGCs [54]. *Igf1r* was positively correlated with the lncRNA *RP23-407B16.5* (Additional file 7: Table S7). Another regeneration-associated gene correlated with lncRNAs in our analysis was *Akt3* [55], a gene downregulated in the injured ooDSGCs. *Akt3* was positively correlated with the lncRNAs *RP23-22A15.2*, *RP23-201 L15.5*, *RP23-256O22.3*, *RP23-128 M23.2*, *XLOC_021844* and *XLOC_048151* (Additional file 7: Table S7). We note however, that our assumptions on the functional roles of these lncRNAs remain speculative, and whether they in fact act to regulate gene expression, RGC specification, and survival remains to be determined.

## Conclusions

In summary, our study has provided identification of lncRNAs expressed in mouse RGC types that are molecularly, physiologically, and functionally distinct from each other. The data from this study could form the foundation for further exploration of lncRNAs and their potential as regulators of retinal cell development and survival after injury.

## Supplementary Information

**Additional file 1: Table S1.** List of primers used in this study.

**Additional file 2: Table S2.** List of lncRNAs detected in ipRGCs and ooDSGCs.

**Additional file 3: Table S3.** RGC type- and injury- specificity of lncRNAs and mRNAs.

**Additional file 4: Table S4.** List of the lncRNAs detected in normal ipRGCs or in normal ooDSGCs that were detected or not detected in the whole retina.

**Additional file 5: Table S5.** List of the differentially expressed lncRNAs detected from various RGC group comparisons.

**Additional file 6: Table S6.** List of correlated lncRNA-nearby protein-coding genes pairs (r > |0.5|).

**Additional file 7: Table S7.** List of lncRNAs with expression correlated to protein coding genes (r > |0.88|).

**Additional file 8: Table S8.** Top GO “Biological Process” and “Molecular Function” enriched terms [*p*-value < 0.05) assigned to the protein-coding genes correlated with lncRNA (r > |0.88|) in each cluster.

**Additional file 9: Fig. S1** Subclasses of lncRNAs detected in ipRGCs and ooDSGCs. Venn diagram showing antisense lncRNAs (AS lncRNAs) (**a**), long intergenic noncoding RNAs (lincRNAs) [**b**] and novel lincRNAs (**c**) expressed under normal and injury (i.e. optic nerve crush) conditions. **Fig. S2** RGC type specificity of lncRNAs and protein-coding genes using a fractional expression level (FEL) across normal and injured ipRGCs and ooDSGCs. Relative abundance of the lncRNAs (*n* = 1415) (**a**) and mRNAs (*n* = 13,366) (**b**) expressed in at least one of the four RGC groups. For each sample, transcript abundance is expressed as a fraction of the sum of the expression values detected in all RGC types (FEL) (see Methods for details). (**c**) Maximal expression abundance (log2-TPM) of each lncRNA and protein-coding across the four RGC groups. The right panel shows the expression levels of 478 lncRNAs (top right) and 3735 protein-coding genes (bottom right) that have a maximal expression level within the range bounded by the dashed segments in the left panel ([2–4.32] log2 TPM). (**d**) FELs of lncRNAs and mRNAs expressed at the full TPM expression range and with maximal expression level within the range 4–20 TPM across the four RGC groups were calculated. The percentages of transcripts with an FEL higher or lower than 50% in each class are shown. Higher FEL values indicate higher specificity for RGC types. The observed fractions of RGC type specific lncRNAs are significantly different compared to those of the mRNAs at the full TPM range and at the 4–20 TPM range. (***) Chi-square test, *p* < 0.0001. **Fig. S3** Expression level comparison between the whole retina lincRNAs and lncRNAs detected in normal ipRGCs (**a**) and normal ooDSGCs (**b**). Transcript length (**c**) and number of exons (**d**) comparison between the whole retina lincRNAs and lncRNAs detected in ipRGCs and ooDSGCs. **Fig. S4** Validating expression of a novel lincRNA in RGCs. (**a**) Representative images of retinal sections from Thy1-CFP mice showing GFP and RBPMS immunoreactivity. Nearly all GFP+ cells are RBPMS+, indicating that RGCs are specifically labelled in this mouse line. (**b**) Representative FACS plots of dissociated retinal cells expressing CFP. CFP+ and CFP- cells were collected for RNA extraction and RT-qPCR. (**c**) Level of expression of *Thy1* and *Slc17a6* (markers for RGCs) and *Rho* (marker for rods). CFP+ cells show enrichment of RGC genes compared to CFP- cells. Data from three biological replicates (mean ± SD; * p-value < 0.05; unpaired two-tailed t test). (**d**) RT-PCR result for one randomly selected differentially expressed lincRNA (*XLOC_020964*). *XLOC_020964* was significantly upregulated in injured ooDSGCs compared to normal ooDSGCs. RNA from optic nerve crushed Thy1-CFP mice was used for reverse transcription. Primer pair was designed to amplify a fragment spanning one intron. PCR amplicon was confirmed by Sanger sequencing. **Fig. S5** Co-expression between lncRNAs and their nearby protein-coding genes. Distribution of Pearson correlation coefficient between the differentially expressed (DE) lncRNAs and their nearby DE mRNAs in the RGC comparisons: injured ooDSGCs vs. normal ooDSGCs (**a**), injured ooDSGCs vs. injured ipRGCs (**b**), and normal ooDSGCs vs. normal ipRGCs (**c**). The correlation was measured using ipRGC and ooDSGC expression data from our study and RGC data from a published single cell RNAseq [9]. The Y-axis shows the distance in kilobase (kb) between each lncRNA and their mRNA neighbor. Histograms depicting the frequency distribution for the X and Y-axis are shown at the top and right, respectively. LncRNA- nearby gene pairs with r > |0.5| (vertical red dashed lines) were considered as correlated. The square of the correlation coefficient (r^2^) in the regression linear between Y and X-axis is shown in each panel. **Fig. S6** Heat map showing the expression level of mRNAs that are correlated neighbors (r > |0.5|) of the differentially expressed lncRNAs. (**a**) Injured ooDSGCs vs. normal ooDSGCs. (**b**) Injured ooDSGCs vs. injured ipRGCs. (**c**) Normal ooDSGCs vs. normal ipRGCs. Expression values were based on Z-score normalized TPM for each mRNA. **Fig. S7** Heat map displaying the correlation between the lncRNAs and a subset of mRNAs from the cluster 2 from Fig. 5a that were enriched in the Biological Processes terms “intrinsic apoptotic signaling pathway in response to endoplasmic reticulum stress” (**a**) and “positive regulation of transcription from RNA polymerase II promoter in response to endoplasmic reticulum stress” (**b**). LncRNAs in purple and green fonts are upregulated and downregulated, respectively, in the injured ooDSGCs vs. normal ooDSGCs comparison. Red to blue Pearson Correlation scale indicates the degree to which mRNA expression is positively (dark red), negatively (dark blue), or not correlated with the expression of the respective lncRNA.

## Data Availability

All data analyzed during this study are included in this article and its supplementary information files. The RNA-seq data from the injured and normal ipRGCs and ooDSGCs used in this article is deposited under the accession number GEO: GSE115661 [11]. The RNA-seq data from the whole retina was downloaded from Sequence Read Archive (SRA) PRJNA514424 [36].

## Abbreviations

LncRNAs: Long noncoding RNAs
RGCs: Retinal ganglion cells
OoDSGCs: ON-OFF direction-selective RGCs
IpRGCs: Intrinsically photosensitive RGCs
LincRNAs: Long intergenic noncoding RNAs
AS lncRNAs: Antisense long noncoding RNAs
CNS: Central nervous system
DSGCs: Direction selective-RGCs
RNA-seq: RNA-sequencing
FPKM: Fragments Per Kilobase Million
DE: Differential expression/ differentially expressed
GO: Gene ontology
RT-PCR: Reverse transcription PCR
ON-OFF: Light onset and offset
