# Supplementary material for: Identification of long noncoding RNAs in injury-resilient and injury-susceptible mouse retinal ganglion cells
Source: BMC Genomics. 2021 Oct 14;22:741. doi: 10.1186/s12864-021-08050-x (PMC8518251; doi:10.1186/s12864-021-08050-x)
Supplement: Supplementary file 1 — Additional file 1: Table S1. List of primers used in this study. [file 12864_2021_8050_MOESM1_ESM.pdf]

**Table S1** List of primers used in this study.

| **Primer Name** | **Oligonucleotides Sequence** |
|---|---|
| Thy1_F | GGGCGACTACTTTTGTGAGC |
| Thy1_R | TCTGAACCAGCAGGCTTATG |
| Slc17a6_F | CATTGGAGAGAGCGCAAATC |
| Slc17a6_R | GTTGGCAACAATTATCGCGT |
| Rho_F | AAGCAGCCTTGGTCTCTGTC |
| Rho_R | AGGGCGATTTCACCTCCAAG |
| Hprt_F | GGGATTTGAATCACGTTTGTG |
| Hprt_R | CAGGACTCCTCGTATTTGCA |
| XLOC_020964_F | AAGGGATGATTCAGCCTGTG |
| XLOC_020964_R | TGCTCCTCTTCTAACAGAACTTGA |
