# Supplementary material for: Identification of long noncoding RNAs in injury-resilient and injury-susceptible mouse retinal ganglion cells
Source: BMC Genomics. 2021 Oct 14;22:741. doi: 10.1186/s12864-021-08050-x (PMC8518251; doi:10.1186/s12864-021-08050-x)
Supplement: Supplementary file 3 — Additional file 3: Table S3. RGC type- and injury- specificity of lncRNAs and mRNAs. [file 12864_2021_8050_MOESM3_ESM.pdf]

Table S3 RGC type- and injury- specificity of lncRNAs and mRNAs.

**Transcript type**	**# Transcripts detected exclusively in ipRGCs**	**# Transcripts detected exclusively in normal ipRGCs**	**# Transcripts detected exclusively in injured ipRGCs**	**# Total transcripts detected in ipRGCs**	**# Transcripts detected exclusively in ooDSGCs**	**# Transcripts detected exclusively in normal ooDSGCs**	**# Transcripts detected exclusively in injured ooDSGCs**	**# Total transcripts detected in ooDSGCs**
lncRNAs	268 (31% of the total)	294 (35% of the total)	188 (22% of the total)	851	564 (49% of the total)	311 (27% of the total)	293 (26% of the total)	1,147
mRNAs	605 (5% of the total)	895 (7% of the total)	526 (4% of the total)	12,229	1,137 (9% of the total)	505 (4% of the total)	855 (7% of the total)	12,761
