# Supplementary material for: Identification of long noncoding RNAs in injury-resilient and injury-susceptible mouse retinal ganglion cells
Source: BMC Genomics. 2021 Oct 14;22:741. doi: 10.1186/s12864-021-08050-x (PMC8518251; doi:10.1186/s12864-021-08050-x)
Supplement: Supplementary file 9 — Additional file 9: Fig. S1 Subclasses of lncRNAs detected in ipRGCs and ooDSGCs. Venn diagram showing antisense lncRNAs (AS lncRNAs) (a), long intergenic noncoding RNAs (lincRNAs) [b] and novel lincRNAs (c) expressed under normal and injury (i.e. optic nerve crush) conditions. Fig. S2 RGC type specificity of lncRNAs and protein-coding genes using a fractional expression level (FEL) across normal and injured ipRGCs and ooDSGCs. Relative abundance of the lncRNAs (n = 1415) (a) and mRNAs (n = 13,366) (b) expressed in at least one of the four RGC groups. For each sample, transcript abundance is expressed as a fraction of the sum of the expression values detected in all RGC types (FEL) (see Methods for details). (c) Maximal expression abundance (log2-TPM) of each lncRNA and protein-coding across the four RGC groups. The right panel shows the expression levels of 478 lncRNAs (top right) and 3735 protein-coding genes (bottom right) that have a maximal expression level within the range bounded by the dashed segments in the left panel ([2–4.32] log2 TPM). (d) FELs of lncRNAs and mRNAs expressed at the full TPM expression range and with maximal expression level within the range 4–20 TPM across the four RGC groups were calculated. The percentages of transcripts with an FEL higher or lower than 50% in each class are shown. Higher FEL values indicate higher specificity for RGC types. The observed fractions of RGC type specific lncRNAs are significantly different compared to those of the mRNAs at the full TPM range and at the 4–20 TPM range. (***) Chi-square test, p < 0.0001. Fig. S3 Expression level comparison between the whole retina lincRNAs and lncRNAs detected in normal ipRGCs (a) and normal ooDSGCs (b). Transcript length (c) and number of exons (d) comparison between the whole retina lincRNAs and lncRNAs detected in ipRGCs and ooDSGCs. Fig. S4 Validating expression of a novel lincRNA in RGCs. (a) Representative images of retinal sections from Thy1-CFP mice showing GFP and R [file 12864_2021_8050_MOESM9_ESM.pdf]

Supplementary Figures

Identification of Long Noncoding RNAs in Injury-Resilient and Injury-Susceptible Mouse Retinal Ganglion Cells

Authors: Ana C. Ayupe[1*], Felipe Beckedorff[2], Konstantin Levay[1], Benito Yon[1], Yadira Salgueiro[1], Ramin Shiekhattar[2], Kevin K. Park[1*]

Affiliation:

[1]Miami Project to Cure Paralysis, Department of Neurosurgery, University of Miami Miller School of Medicine, 1095 NW 14th Ter. Miami, FL 33136

[2]University of Miami Miller School of Medicine, Sylvester Comprehensive Cancer Center, Department of Human Genetics, Biomedical Research Building, Room 719, 1501 NW 10th Avenue, Miami, FL 33136, USA

*Corresponding Authors: Kevin K. Park, kpark@miami.edu; Ana C. Ayupe, aca136@med.miami.edu

Figure S1

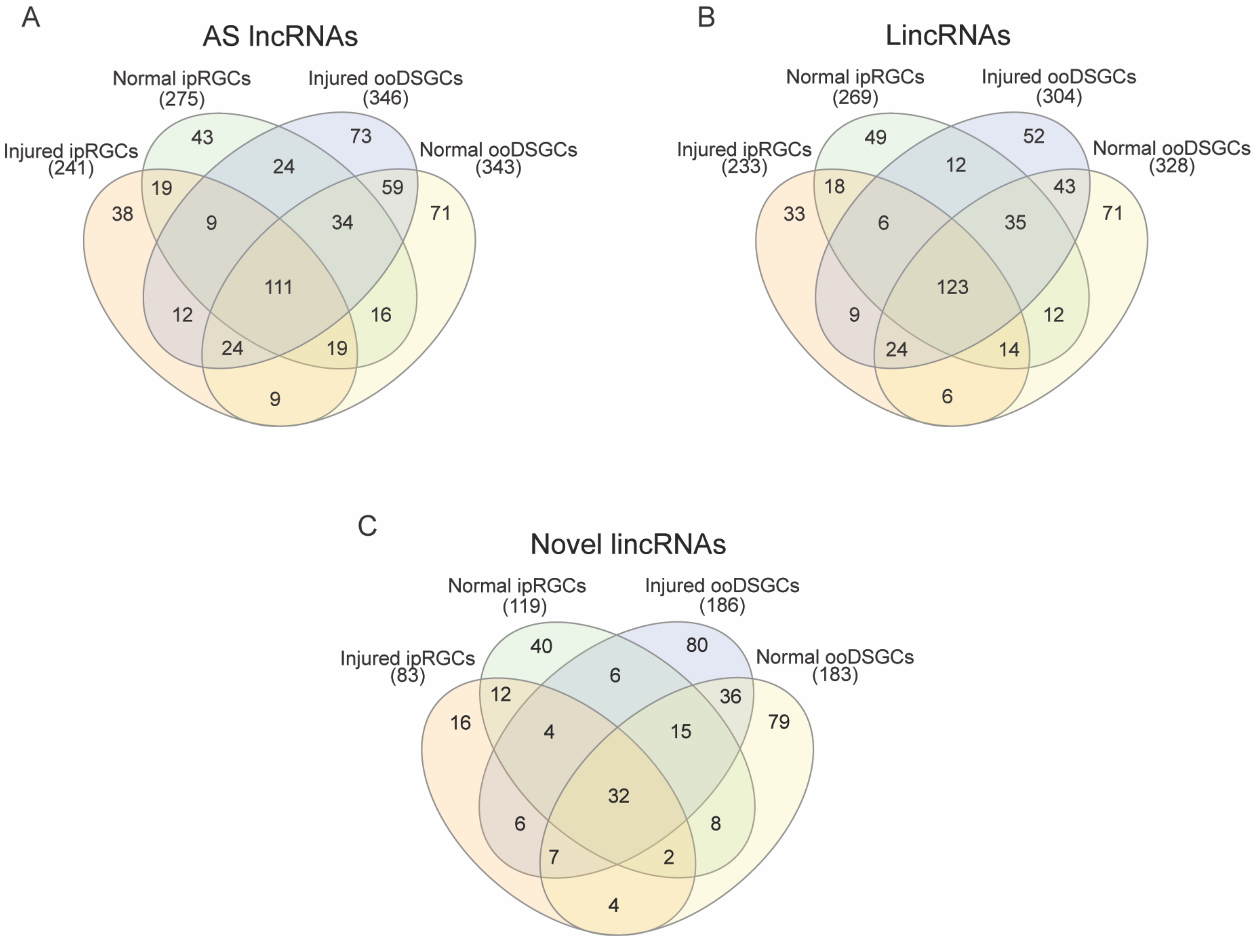
A
AS lncRNAs
Normal ipRGCs
(275)
Injured ooDSGCs
(346)
Injured ipRGCs
(241)
Normal ooDSGCs
(343)
43
73
24
19
59
38
9
34
71
111
12
16
24
19
9
B
LincRNAs
Normal ipRGCs
(269)
Injured ooDSGCs
(304)
Injured ipRGCs
(233)
Normal ooDSGCs
(328)
49
52
12
18
43
33
6
35
71
123
9
12
24
14
6
C
Novel lincRNAs
Normal ipRGCs
(119)
Injured ooDSGCs
(186)
Injured ipRGCs
(83)
Normal ooDSGCs
(183)
40
80
6
12
36
16
4
15
79
32
6
8
7
2
4

Figure S2

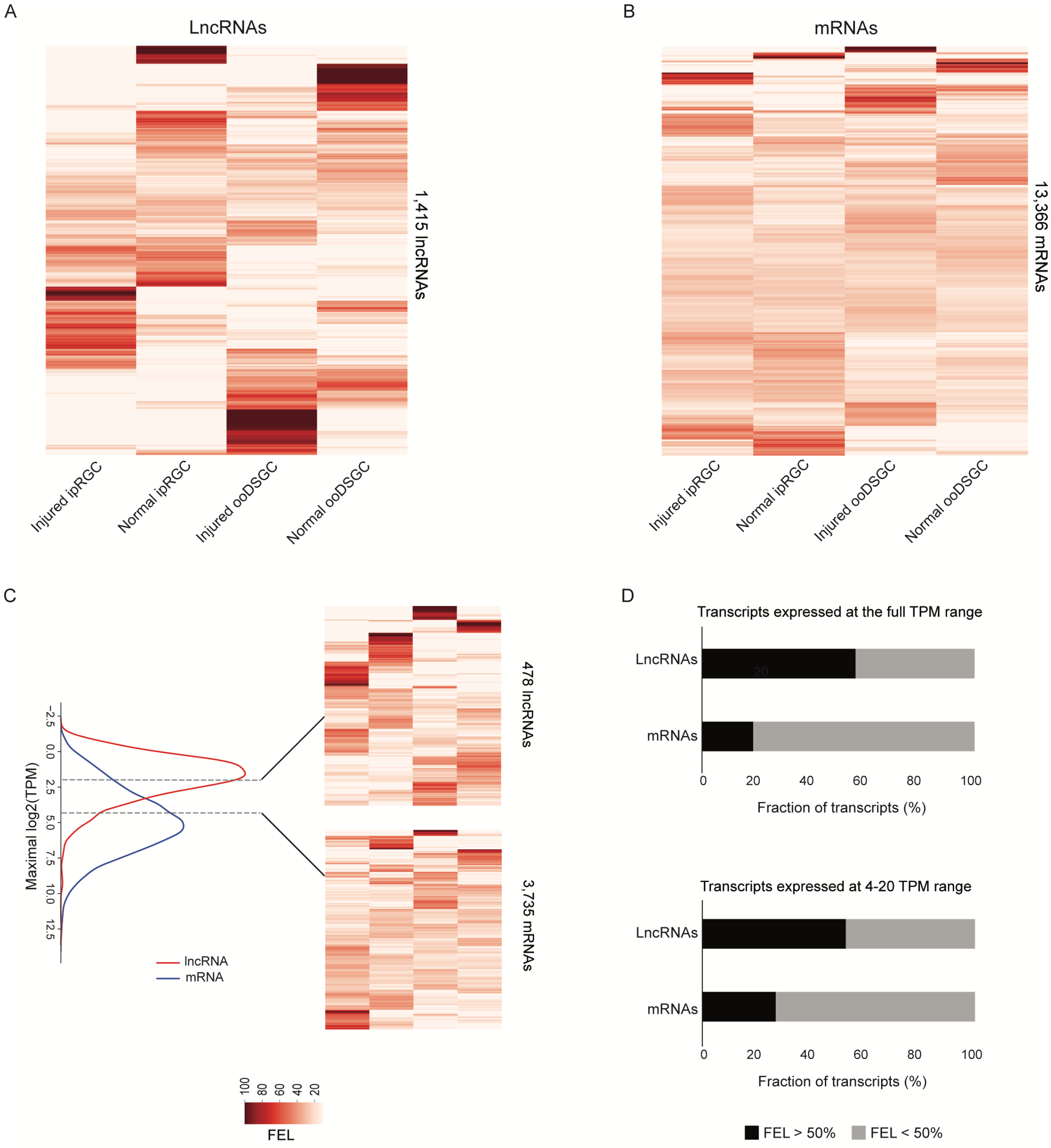
A
LncRNAs
1,415 lncRNAs
Injured ipRGC
Normal ipRGC
Injured ooDSGC
Normal ooDSGC
B
mRNAs
13,366 mRNAs
Injured ipRGC
Normal ipRGC
Injured ooDSGC
Normal ooDSGC
C
Maximal log2(TPM)
-2.5
0.0
2.5
5.0
7.5
10.0
12.5
lncRNA
mRNA
478 lncRNAs
3,735 mRNAs
100
80
60
40
20
FEL
D
Transcripts expressed at the full TPM range
LncRNAs
mRNAs
0
20
40
60
80
100
Fraction of transcripts (%)
Transcripts expressed at 4-20 TPM range
LncRNAs
mRNAs
0
20
40
60
80
100
Fraction of transcripts (%)
FEL > 50%
FEL < 50%

Figure S3

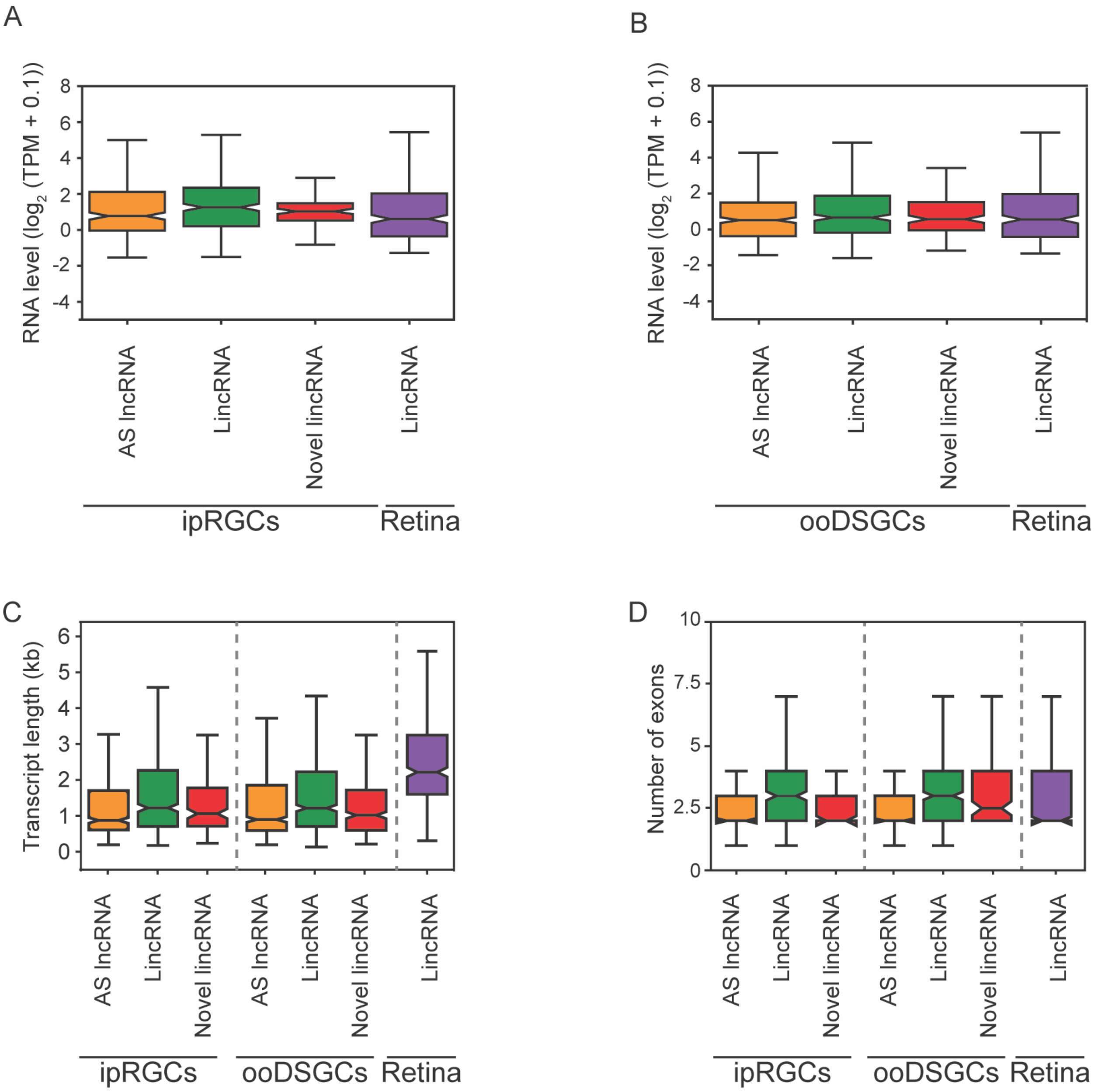
A
RNA level (log2 (TPM + 0.1))
8
6
4
2
0
-2
-4
AS lncRNA
LincRNA
Novel lincRNA
LincRNA
ipRGCs
Retina
B
RNA level (log2 (TPM + 0.1))
8
6
4
2
0
-2
-4
AS lncRNA
LincRNA
Novel lincRNA
LincRNA
ooDSGCs
Retina
C
Transcript length (kb)
6
5
4
3
2
1
0
AS lncRNA
LincRNA
Novel lincRNA
AS lncRNA
LincRNA
Novel lincRNA
LincRNA
ipRGCs
ooDSGCs
Retina
D
Number of exons
10
7.5
5
2.5
0
AS lncRNA
LincRNA
Novel lincRNA
AS lncRNA
LincRNA
Novel lincRNA
LincRNA
ipRGCs
ooDSGCs
Retina

Figure S4

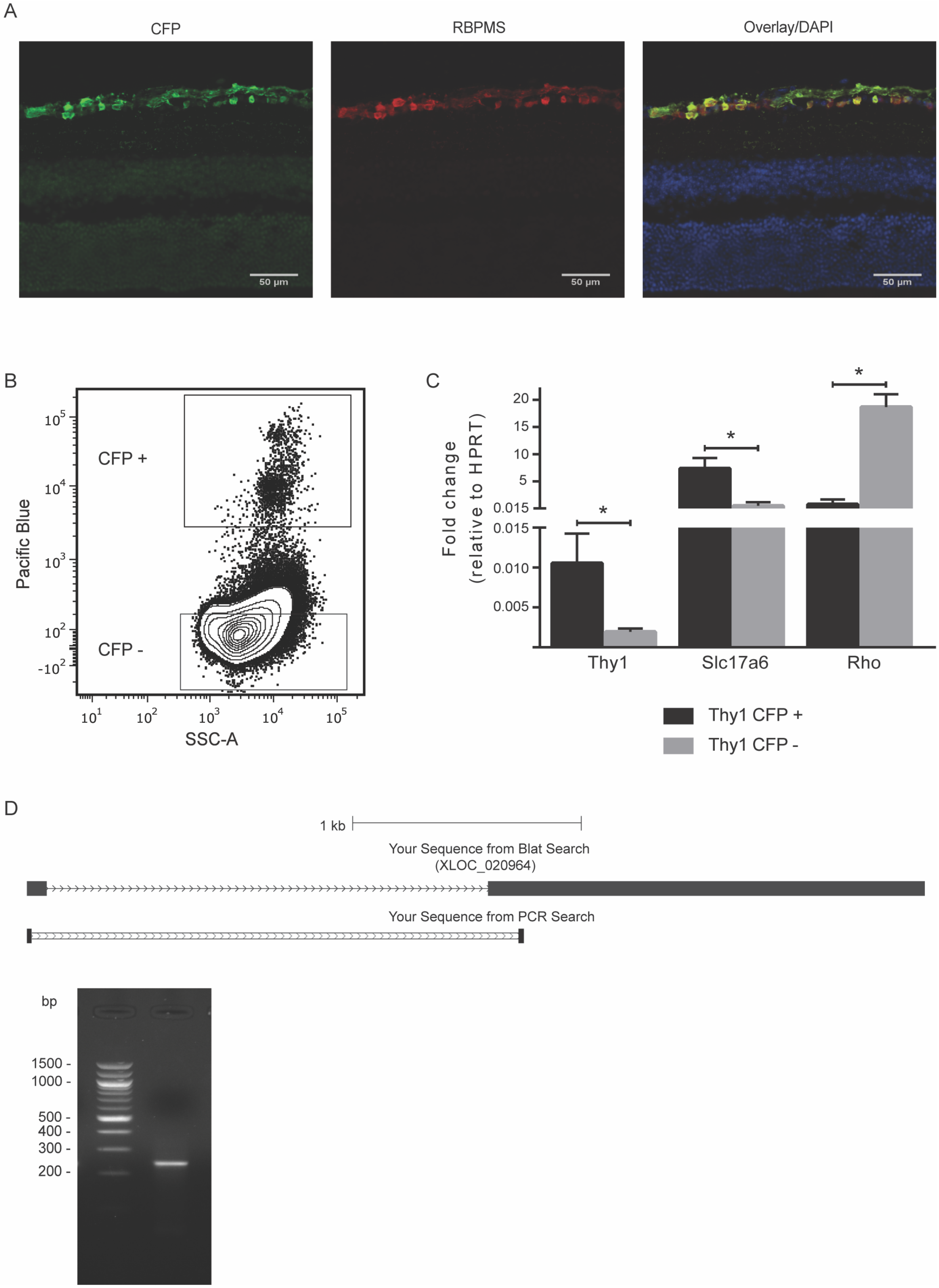
A
CFP
RBPMS
Overlay/DAPI
50 µm
B
CFP +
CFP -
Pacific Blue
SSC-A
C
Fold change
(relative to HPRT)
20
15
10
5
0.015
0.010
0.005
Thy1
Slc17a6
Rho
Thy1 CFP +
Thy1 CFP -
D
1 kb
Your Sequence from Blat Search
(XLOC_020964)
Your Sequence from PCR Search
bp
1500 -
1000 -
500 -
400 -
300 -
200 -

Figure S5

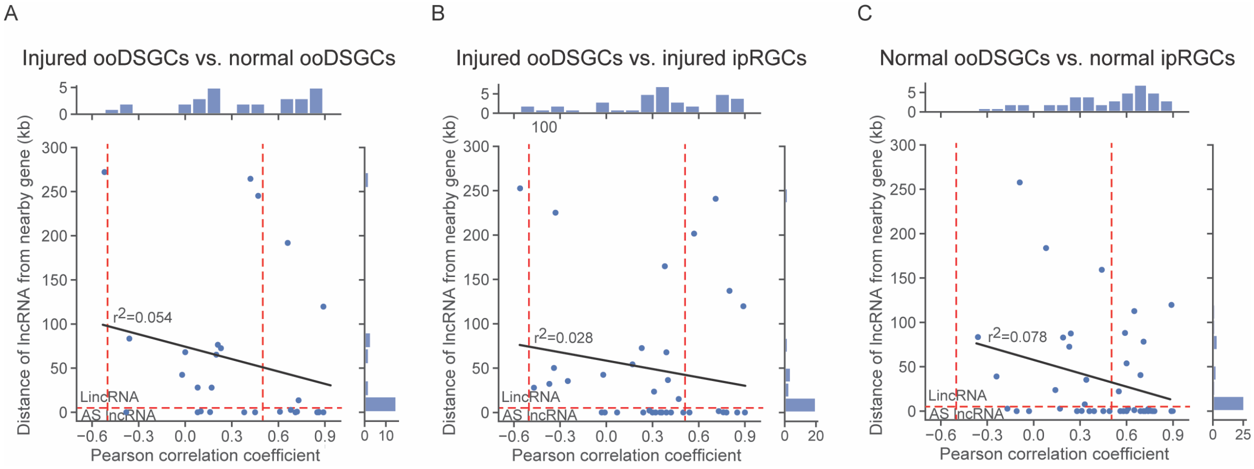
A
Injured ooDSGCs vs. normal ooDSGCs
r2=0.054
LincRNA
AS lncRNA
Distance of lncRNA from nearby gene (kb)
Pearson correlation coefficient
B
Injured ooDSGCs vs. injured ipRGCs
r2=0.028
C
Normal ooDSGCs vs. normal ipRGCs
r2=0.078

Figure S6

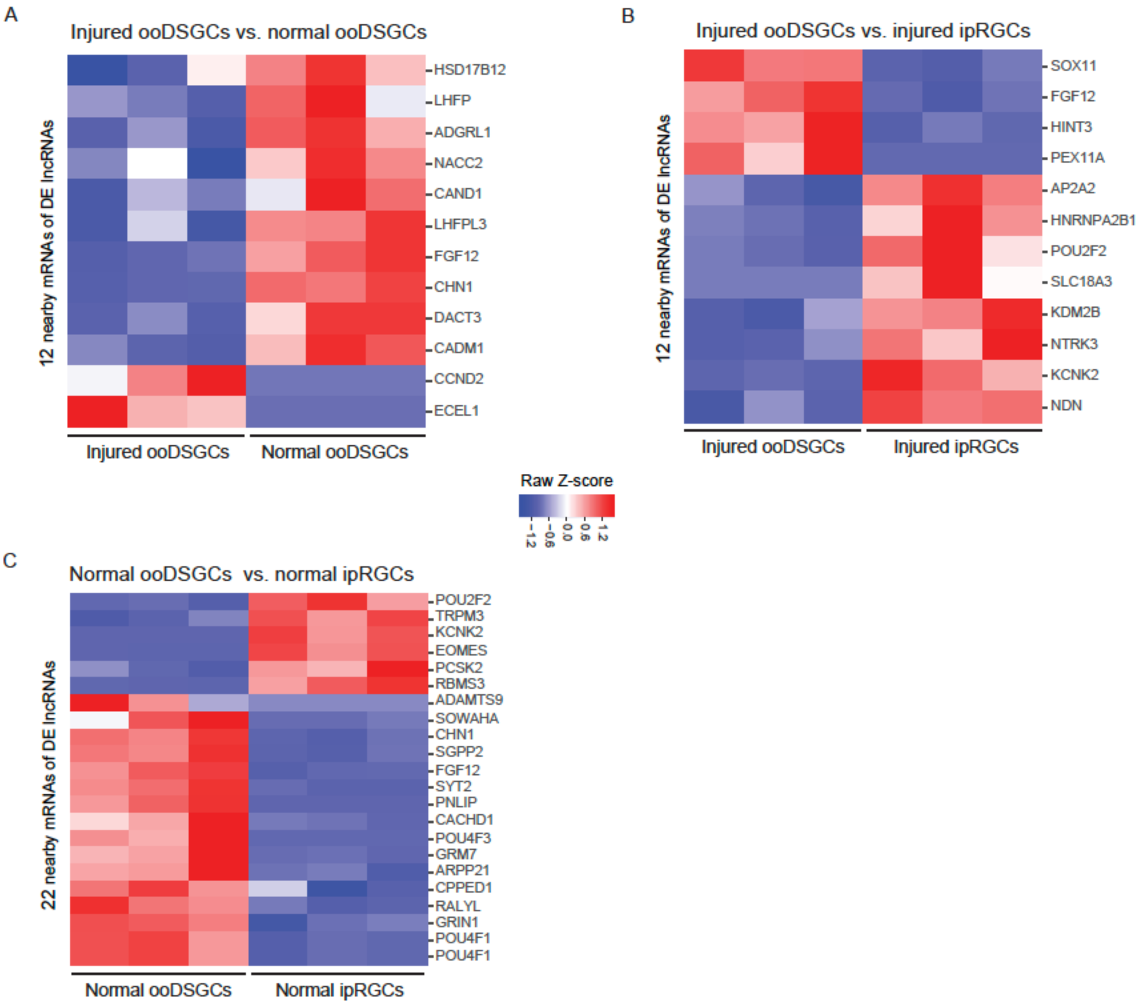
A
Injured ooDSGCs vs. normal ooDSGCs
12 nearby mRNAs of DE lncRNAs
HSD17B12
LHFP
ADGRL1
NACC2
CAND1
LHFPL3
FGF12
CHN1
DACT3
CADM1
CCND2
ECEL1
Injured ooDSGCs
Normal ooDSGCs
B
Injured ooDSGCs vs. injured ipRGCs
12 nearby mRNAs of DE lncRNAs
SOX11
FGF12
HINT3
PEX11A
AP2A2
HNRNPA2B1
POU2F2
SLC18A3
KDM2B
NTRK3
KCNK2
NDN
Injured ooDSGCs
Injured ipRGCs
Raw Z-score
-1.2
-0.6
0.0
0.6
1.2
C
Normal ooDSGCs vs. normal ipRGCs
22 nearby mRNAs of DE lncRNAs
POU2F2
TRPM3
KCNK2
EOMES
PCSK2
RBMS3
ADAMTS9
SOWAHA
CHN1
SGPP2
FGF12
SYT2
PNLIP
CACHD1
POU4F3
GRM7
ARPP21
CPPED1
RALYL
GRIN1
POU4F1
POU4F1
Normal ooDSGCs
Normal ipRGCs

Figure S7

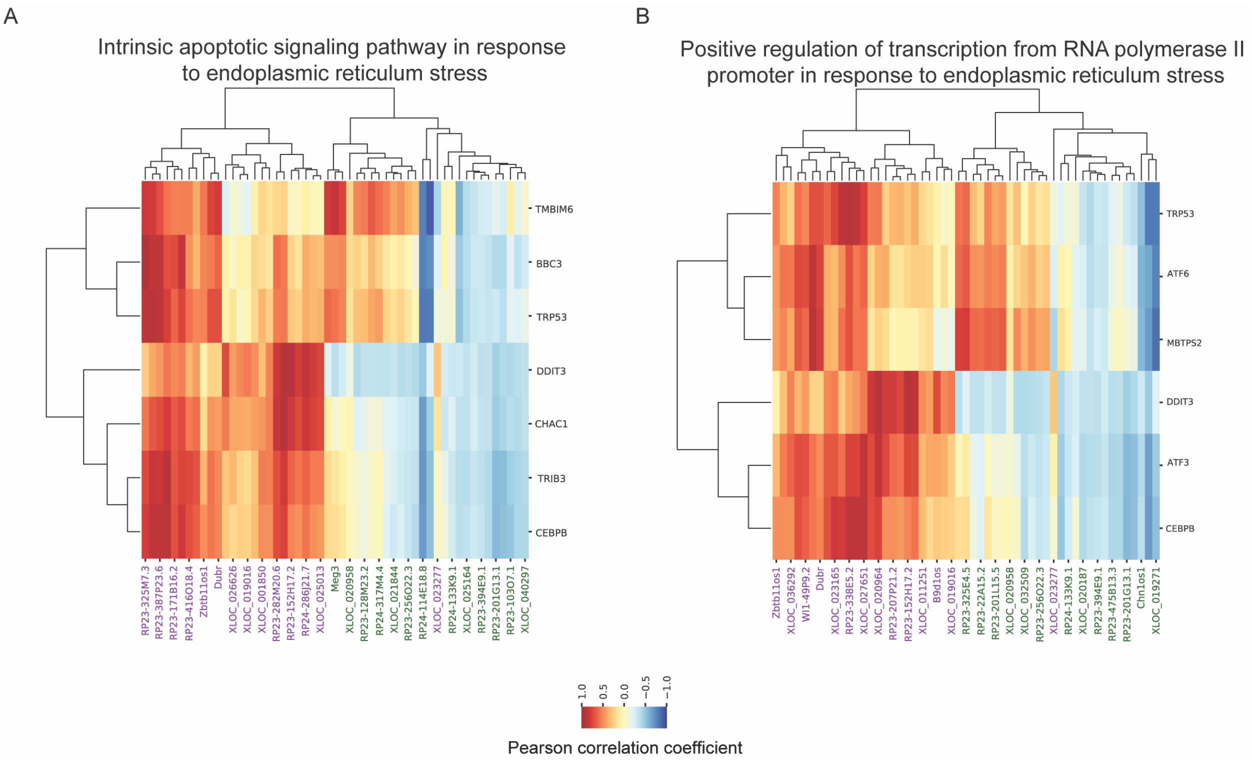
A
Intrinsic apoptotic signaling pathway in response to endoplasmic reticulum stress
TMBIM6
BBC3
TRP53
DDIT3
CHAC1
TRIB3
CEBPB
RP23-325M7.3
RP23-387P23.6
RP23-171B16.2
RP23-416O18.4
Zbtb11os1
Dubr
XLOC_026626
XLOC_019016
XLOC_001850
RP23-282M20.6
RP23-152H17.2
RP24-286J21.7
XLOC_025013
Meg3
XLOC_020958
RP23-128M23.2
RP24-317M4.4
XLOC_021844
RP23-256O22.3
RP24-114E18.8
XLOC_023277
RP24-133K9.1
XLOC_025164
RP23-394E9.1
RP23-201G13.1
RP23-103O7.1
XLOC_040297
B
Positive regulation of transcription from RNA polymerase II promoter in response to endoplasmic reticulum stress
TRP53
ATF6
MBTPS2
DDIT3
ATF3
CEBPB
Zbtb11os1
XLOC_036292
Wl1-49P9.2
Dubr
XLOC_023165
RP23-338E5.2
XLOC_027651
XLOC_020964
RP23-207P21.2
RP23-152H17.2
XLOC_011251
B9d1os
XLOC_019016
RP23-325E4.5
RP23-22A15.2
RP23-201L15.5
XLOC_020958
XLOC_032509
RP23-256O22.3
XLOC_023277
RP24-133K9.1
XLOC_020187
RP23-394E9.1
RP23-475B13.3
RP23-201G13.1
Chn1os1
XLOC_019271
1.0
0.5
0.0
−0.5
−1.0
Pearson correlation coefficient
